# Small interfering RNA targeting of S phase kinase-interacting protein 2 inhibits cell proliferation of pterygium fibroblasts

**Published:** 2011-01-22

**Authors:** Ying Su, Feng Wang, Hu Qi, Shi Guang Zhao, Xue Li, Hao Cui

**Affiliations:** Department of Ophthalmology, First Clinic College of Harbin Medical University, Harbin, China

## Abstract

**Purpose:**

Fibroblast cell proliferation is major reason for recurrence of pterygia. In the present study, we investigated if small interfering RNA (siRNA)-mediated gene silencing of S phase-kinase-interacting protein 2 (*Skp2*) can be employed to inhibit protein 27 kinase inhibition protein 1 (*p27^kip1^*) down-regulation in pterygium fibroblast cells (PFC) in vitro and in vivo.

**Methods:**

A plasmid containing transgenes encoding *Skp2* siRNA was used to decreasing the high constitutive levels of Skp2 protein in PFC and normal fibrboblast cells (NFC) in vitro and in vivo which can lead to consequent degradation of *p27^kip1^*. Cell proliferation and viability were investigated using cell counts, 59-bromodeoxyuridine incorporation (BrdU assay) and tetrazolium reduction (MTT assay).

**Results:**

Infection of PFC and NFC with *Skp2* siRNA resulted in significant inhibition of cell proliferation and metabolic activity in vitro. Immunoflurescence showed decreased levels of Skp2 and increased levels of p27^kip1^ in *pSkp2* siRNA infected cells, but not in plasmid and uninfected cells.

**Conclusions:**

*Skp2* siRNA inhibited the cell proliferation of PFC in vitro and in vivo.

## Introduction

Pterygium is a common proliferative disorder involving epithelial hyperplasia and fibrovascular proliferation. In the advanced stages, pterygium can necessitate complex surgery for full visual rehabilitation and recurrences, and ocular complications are not infrequent [1-3]. Ultraviolet (UV) light has long been regarded as the etiologic agent of pterygium [4-6].

The etiology and physiopathology of pterygium have not been completely defined. However, it is well known from epidemiologic and histologic studies that the most important factor in its appearance and development is sunlight exposure. This causes proliferation of fibrovascular tissue which invades the cornea from the exposed conjunctiva.

Although the pathogenesis of pterygium is yet undetermined, tumor-like histologic characteristics, ranging from mild dysplasia to carcinoma in situ and local invasiveness, have been described by different authors [7-9]. Moreover, pterygium fibroblasts (PFs) exhibit characteristics of the transformed phenotype [10], microsatellite instability, and loss of heterozygosity, all reported to be common findings in neoplastic tissue.

However, the mechanisms in the regulation of lens cell proliferation are still unclear.

S phase-kinase-interacting protein 2 (Skp2) was identified as the E3 ubiquitin ligase that targets protein 27 kinase inhibition protein 1 (p27^kip1^) for ubiquitination [11-13]. Skp-cullin-F (SCF) complexes represent an evolutionarily conserved class of E3 enzymes containing four subunits: Skp1, cullin 1 (Cul1), one of many F box proteins, and ring box protein 1 (Rbx1)  [14]. Skp2, an F box protein, is required for the ubiquitination and consequent degradation of p27 both in vivo and in vitro. Skp2 is specifically required for p27 ubiquitination and that Skp2 is a rate-limiting component of the machinery that ubiquitinates and degrades phosphorylated p27. Skp2 is frequently overexpressed in tumor cell lines, and forced expression of Skp2 in quiescent fibroblasts induces DNA synthesis [15].

We showed that expression of Skp2 can be detected in rabbit tenon’s fibroblast cells and confirmed that transfection of *Skp2* siRNA can effectively inhibit the proliferation of rabbit tenon’s fibroblast cells after glaucoma surgery [16].

In this study, we examined the expression of Skp2 in pterygium fibroblast cells (PFC) and investigated if small interfering RNA (siRNA)-mediated gene silencing of *Skp2* can be employed to inhibit protein 27 kinase inhibition protein 1 (*p27^kip1^*) down-regulation in PFC and the effects of down-regulation of *p27^kip1^* on PFC proliferation.

## Methods

### Patient selection

We recruited eight patients with primary pterygium (age range, 42–66 years; mean, 55.32±2.3 years) and eight eyes with normal conjunctiva (age range, 45–65 years; mean, 56.25±1.5 years) which were donated. Diagnosis of pterygium was based on clinical history and evaluation of signs and symptoms. All patients with pterygium (five men and three women) had at least a five-year history of a slow-growing lesion, with a corneal extension of at least 4 mm, as measured with a caliper, from the limbus to the corneal vertex. Pterygia morphology was clinically graded based on the assessment of pterygium translucency: atrophic (T1), intermediate (T2), or fleshy (T3) pterygium. All pterygia collected in this study were T3. No subject in the control group had any inflammatory signs or symptoms. The research adhered to the tenets of the Declaration of Helsinki. Written informed consent was obtained from all the patients before tissues were collected. This study and all the procedures ere approved by the Ethics Committee of the University of Harbin Medical University. All tissue samples were obtained as previously described [5]. In summary, at the time of surgery, tissue was excised from the inner canthus with microforceps. As a rule, within 1 h of excision, samples were placed in sterile boxes containing a preservative solution and were transferred to the cell culture room.

PlasmidSuppressor (pSuppressor) containing *Skp2* siRNA and control pSuppressor was purchased from Imgenex (San Diego, CA). Synthetic oligonucleotide primers was purchased from Qiagen (Valencia, CA). Skp2 antibody and the Steptavidin-Alkaline Phosphatase Complex (SABC) kit were purchased from the Boster Company (Wuhan, China). Enhanced chemiluminescence kit, p27^kip1^ antibody, and PCNA antibody were purchased from Santa Cruz Biotechnology, Inc. (Santa Cruz, CA). Fluorescein-conjugated anti-sheep IgG was purchased from Zhongshan Biotechnology (Beijing, China). Vectashield mounting medium was purchased from Vector laboratories (Burlington, CA). Dulbecco’s modified Eagle’s medium and F12 medium (DMEM F12) was purchased from Gibco (Burlington, VT). Streptomycin, penicillin, fetal calf serum and Trizol™ were purchased from Invitrogen (Carlsbad, CA). Hanks solution was purchased from Hyclone (Logan, UT). Poly-lysine was purchased from Sigma (St. louis, MO). Culture plate was purchased from BD Biosciences (San Jose, CA). Hiperfect transfection reagent was purchased from Qiagen. Methylthiazolyltetrazolium (MTT) was purchased from Sigma. MTT cell proliferation kit was purchased from the ATCC (Manassas, VA). The Cell Counter was purchased from Coulter Z1 (Coulter, FL). Metafectene Pro reagent was purchased from Biontex (Martinstried, Germany).

### Equipment

The fluorescence microscope (IX70; Olympus, Tokyo, Japan), optic microscope (Olympus, Tokyo, Japan), CO_2_ incubator (BB16HF: Heal Force, Hong Kong, China), ultraclean work Table (D8C-010; Heal Force), and incubation plate (Costar Corporation, Cambridge, MA).

### Cell culture of human pterygium fibroblasts (hPF)

For fibroblast isolation, pterygium and normal conjunctiva tissues were dissected into three to four tissue fragments (1 mm^2^). The planted tissue was attached to the bottom of a six-well plate with a sterile coverslip and overlaid with DMEM medium. All culture media were supplemented with streptomycin (50 mg and penicillin 500,000 U/l) . To ensure fibroblast cell proliferation, the media was also supplemented with fetal calf serum (FCS; 10% of final volume). Cells from passage 3–5 were used for all experiments. All cells were grown at 37 °C with 5% CO_2_ ventilation.

### Plasmids and transfection

Vector pSuppressorNeo generates biologically active siRNAs from the U6promoter. Synthetic oligonucleotide primers (5’-tcg aGG GAG UGA CAA AGA CUU UGg agu acu gCA AAG UCU UUG UCA CUC CCU UUU U-3’ and 5’-cta gAA AAA GGG AGU GAC AAA GAC UUU Gca gua cuc CAA AGU CUU UGU CAC UCC C-3’) containing XhoI and XbaI overhangs were annealed and then were introduced into pSuppressor Neovector. Oligo-nucleotide sequences correspond to a 19-nucleotide sequence from Skp2 (nucleotides114–133), which is separated by an 8-nucleotide linker from the reverse complement of the same 19-nucleotide sequence. A transcriptional termination (UUUUU) was added to the end of oligonucleotide. We used circular control plasmid, which contains a scrambled sequence as a control. Human ptergium fibroblasts (hPF) transfected with pSuppressor containing *Skp2* siRNA, pSuppressor only, and medium served as experimental group, vehicle control group, and blank control group, respectively. Transfection was performed in 60 mm plates using 2 μg of vector in 10 μl of Metafectene Pro reagent. After 48 h of transfection, cells were treated with G418 for 2 weeks for positive clone selection. After G418 treatment, we cloned several stably transfectant cells. Each clone was screened for expression of Skp2 by western blot analysis.

### Immunocytochemistry

Cells cultured on coverslips were fixed with 4% paraformaldehyde for 10 min at room temperature, permeabilized, and blocked with 0.1% Triton X-100 and 5% goat serum for 30 min. Cells were incubated with rabbit anti-Vimentin antibody (1:500) at 4 °C overnight, followed by goat-anti-rabbit IgG (1:500) for 1 h at room temperature. Then, cells were incubated with strep-avidin-biotin complex (SABC) at 37 °C for 30 min and colored with DAB. After coloration with DAB, dehydration, and dimethyl benzene treatment, slides were mounted. Controls were stained by omitting the primary antibody.

Cells were incubated with Skp2 monoclonal antibody (1:500 dilution), p27^kip1^ antibody (1:500 dilution), PCNA antibody (1:1,000 dilution) at 4 °C overnight, followed by secondary antibody for immunoflurescence staining, respectively. Controls were stained by omitting the primary antibody.

#### (3-(4,5-dimethylthiazolyl-2-)-2,5-diphenyltetrazoliumbromide, MTT) cell viability assay

Cell viability was examined by the MTT cell proliferation kit following the instructions of the manufacturer. The assay is based on measuring the reduction of yellow tetrazolium to a purple for mazanas facilitated by dehydrogenases of metabolically active cells. The intracellular for mazan can be solubilized and quantified by spectrophotometric means. Quadruple samples of hPFC were grown on 96 well plates and were infected with 100 mol of either of the two vectors, or weren’t infected. After 2, 4, 6, 8, and 14 days, wells were incubated in a medium containing yellow tetrazolium for 20 h.

#### Cell proliferation and bromodeoxyuridine (BrdU) incorporation

Cells (5.0×10^3^) were plated onto a 24-well multiwell plate (3047, Falcon; Becton Dickinson, Franklin Lakes, NJ) and allowed to attach for 24 h. The culture medium was then replaced with fresh medium. Then, trypsinized cells were counted suing a cell counter (Coulter Z1; Coulter) at 0, 2, 4, and 6 days. For bromodeoxyuridine (BrdU) incorporation, cells growing on coverslips were incubated with 10 μmol/l BrdU for 3 h. The cells were fixed in cold methanol/acetone 1:1 for 10 min. In brief the cells were sequentially incubated in 1.5 mol/l HCl for 10 min. Then cells were washed thrice with PBS and incubated with the mouse anti-BrdU-primary antibody for 1 h. The cells were washed four times with PBS. The nuclei were simultaneously stained with 10 mg/ml of 4V, 6-diamidino-2-phenylindole. Cells with different BrdU-incorporation patterns were checked and counted with a microscope.

### Animals

Male rabbits (2–3 kg) were supplied by the Experimental Animal Center of Harbin Medical University. All animal care and experimental procedures were in accordance with our institutional guidelines for animal care and the Guide for Care and Use of Laboratory Animals published by the US National Institutes of Health (NIH publication no. 85–23, revised 1996).

### Developing the pterygium model in rabbits and *pSkp2* siRNA delivery

We transplanted an auricular cartilage fragments (2×2 mm) obtained from the same rabbit’s ear. The fragment was grafted onto the upper limbal area of the rabbit’s eye, positioning it between the conjunctiva and the sclera. The pterygium on the rabbit eye formed after the cartilage fragment transplantion for six weeks.

A hypothesis concerning the origin of pterygium suggests that the lachrymal film’s unequal distribution stimulates its growth. We created an area of irregular distribution of tears by inserting cartiliage into the rabbits’ scleral-corneal limbus to evaluate this hypothesis. A slight to moderate inflammatory reaction was observed during the first days in the surgically affected area. The reaction then disappeared, leaving the cartiliage transplanted in the subconjunctival space visible. An initial inflammation reaction was observed, followed by later formation of fibrous tissue in the injured area. Its macroscopic aspect was similar to that of pterygium. Chronic inflammation, fibrosis, an increased number of fibroblasts, and the presence of immune system ploymorphonuclear cells were found; however, no elastosis or hyalinization of subepithelial connective tissue was observed, these being characteristic signs of pterygium.

Vector (2 µg) in 10 µl of Metafectene was delivered to the pterygium by subconjunctival injection at the superonasal quadrant 14 days before the surgery. The pterygium was cut. The neighboring fibroblast and tenon’s capsule fibroblast was cut after transfection during the surgery.

### BrdU incorporation in vivo

After transfection for 14 days, 0.5 ml BrdUrd (10 µmol/l) was injected into the tenon’s capsule fibroblast.

### Pathological and histochemistry examinations

The animals were perfused through the heart with heparin saline followed by 4% paraformaldehyde after 14 day transfection. Eyes were fixed overnight, and transferred to a 30% sucrose solution overnight (4 °C). Frozen sections (5 μm) were cut longitudinally on a cryostat, thaw mounted onto coated glass slides, and stained with hematoxylin and eosin [3], skp2 antibody, p27^kip1^ antibody, PCNA antibody, and anti-Brdu-fluorescein primary antibody. The procedure of histochemistry in vivo was the same as in vitro.

### Statistical analysis

Data were analyzed by the two-tailed Student *t*-test using Origin ver. 6.0 software (OriginLab Corp., Northampton, MA) and a p<0.05 was considered significance.

## Results

### Downregulation of Skp2 protein by siRNA in hPFC

**Figure 1:**
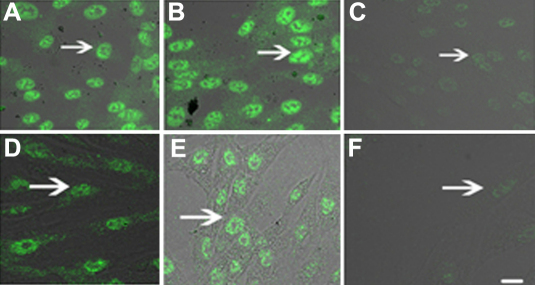
Down-regulation of Skp2 protein by siRNA in PFC in vitro by immunoflurescence. Immunoflurescence staining indicated high constitutive levels of Skp2 protein (arrow indication) in the nucleolus of PFC and NFC transfected with pSuppressor vehicle (**A**, **D**) or without transfection (**B**, **E**). Transfection with *Skp2* siRNA dramatically decreased the expression of Skp2 protein in PFC cells (**C**, **F**). The scale bar is equal to 40 μm.

**Figure 2:**
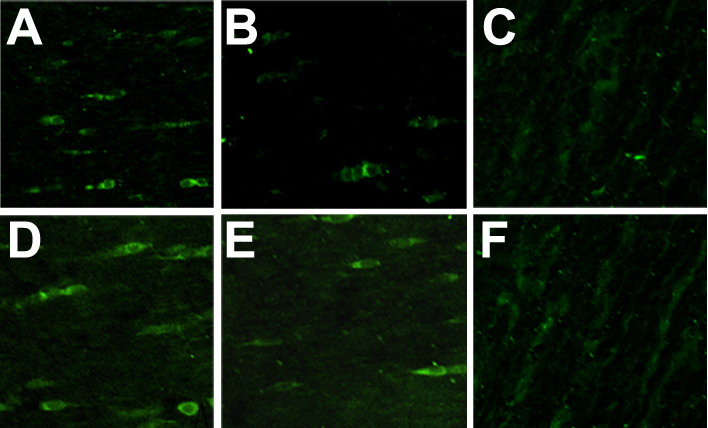
Down-regulation of Skp2 protein by siRNA in PFC in vivo by immunoflurescence. Immunoflurescence staining indicated high constitutive levels of Skp2 protein in the nucleolus of PFC and NFC transfected with pSuppressor vehicle (**A**, **D**) or without transfection (**B**, **E**). Transfection with *Skp2* siRNA dramatically decreased the expression of Skp2 protein in PFC cells (**C**, **F**). The scale bar is equal to 40 μm.

After 48 h of transfection and 2 weeks of treatment with G418, we cloned several stably transfectant cells. Immunoflurescence staining demonstrated high constitutive expression of Skp2 protein in nucleolus of PFC and normal fibroblast cells (NFC) transfected with pSuppressor vehicle (Figure 1A,D and Figure 2A,D) or without transfection (Figure 1B,E and Figure 2B,E). Transfection with *Skp2* siRNA dramatically decreased the expression of Skp2 protein in cells of PFC (Figure 1C,F and Figure 2C,F).

### Upregulation of p27^kip1^ protein by siRNA in hPFC

**Figure 3:**
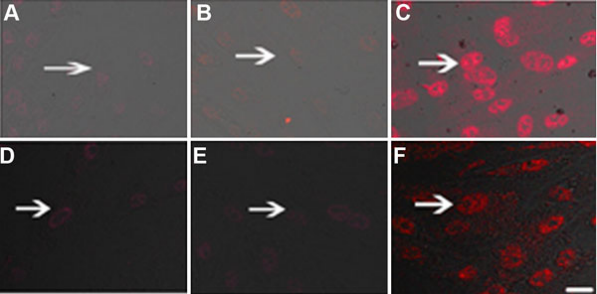
*Skp2* siRNA induced p27^kip1^ accumulationin in PFC in vitro by immunofluorescence. Immunofluorescence staining indicated the expression of p27^kip1^ dramatically increased in PFC and NFC transfection with *Skp2* siRNA (**C**, **F**) when compared with PFC transfection with pSuppressor vehicle (**A**, **D**) or without transfection (**B**, **E**). The scale bar is equal to 40 μm.

**Figure 4:**
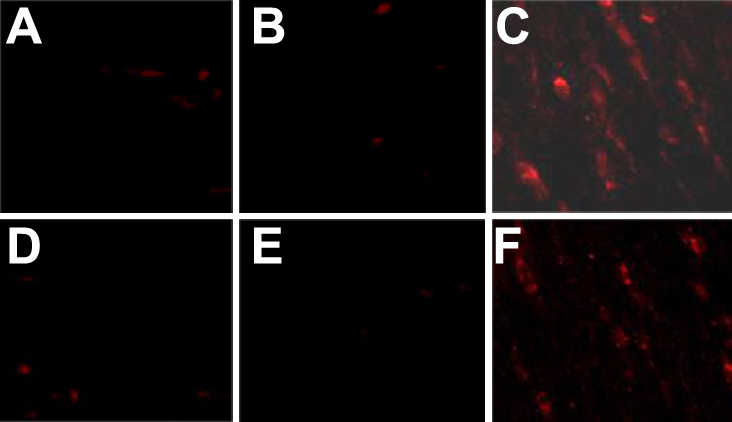
*Skp2* siRNA induced p27^kip1^ accumulationin in PFC in vivo by immunofluorescence. Immunofluorescence staining indicated the expression of p27^kip1^ dramatically increased in PFC and NFC transfection with *Skp2* siRNA (**C**, **F**) when compared with PFC transfection with pSuppressor vehicle (**A**, **D**) or without transfection (**B**, **E**). The scale bar is equal to 40 μm.

Skp2 is required for the ubiquitination and consequent degradation of p27^kip1^ [11]. Immunoflurescence staining demonstrated little expression of p27 protein in the PFC transfected with pSuppressor vehicle (Figure 3A,D and Figure 4A,D) or without transfection (Figure 3B,E and Figure 4B,E) in vitro. Transfection with *Skp2* siRNA increased the expression of p27^kip1^ protein in cells of PFC and NFC (Figure 3C,F and Figure 4C,F) in vitro. Upregulation of p27^kip1^ made it possible for us to investigate the effect of p27^kip1^ inhibition on the proliferation of PFC and NFC.

### *Skp2* siRNA inhibited cell proliferation of PFC in vitro

#### MTT

**Figure 5:**
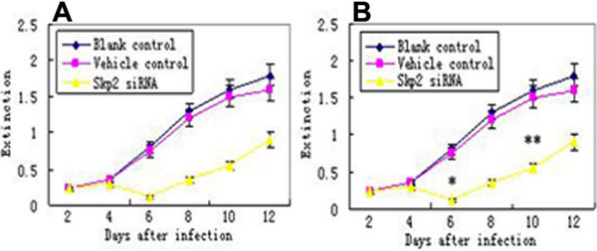
Cell viability assay by MTT. After 6 to 12 days transfection with *Skp2* siRNA, cell viability was significantly decreased in cultured hPFC cells compared with vehicle control and blank control epsecially on the 6th day after transfection. *p<0.01 versus vehicle and blank control; **p<0.05 versus vehicle and blank control.

After transfection, the number of cells increased less in *Skp2* siRNA transfectant hPFC and hNFC than vehicle control and blank control groups (Figure 5A,B).

#### Brdu incorporation

**Figure 6:**
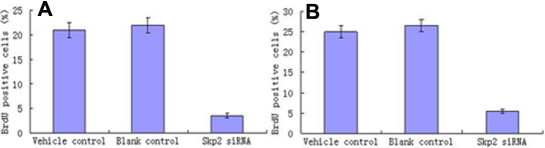
*Skp2* siRNA inhibited the cell proliferation of PFC in vitro (Brdu). For Brdu incorporation, cells growing on coverslips were incubated with Brdu. Incorporated Brdu was detected with antibodies as described in the Methods. Statistical analysis after cell counting showed that Brdu positive cells decreased after hPFC transfection with *Skp2* siRNA compared with control cells. **p<0.01 versus vehicle and blank control.

**Figure 7:**
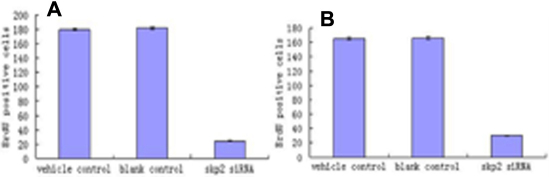
*Skp2* siRNA inhibited the cell proliferation of PFC in vivo (Brdu). For Brdu incorporation, ptergium tissue were incubated with Brdu. Incorporated Brdu was detected with antibodies as described in the Methods. Statistical analysis after cell counting showed that Brdu positive cells decreased after hPFC transfection with *Skp2* siRNA compared with control cells. **p<0.01 versus vehicle and blank control.

For BrdU incorporation assay, cells growing on coverslips were incubated with BrdU. Statically analysis (Figure 6A,B) after cell counting showed that Brdu positive cells in hPF and hNFC transfected with *Skp2* siRNA significantly decreased compared with control cells (p<0.01 versus vehicle and blank control; Figure 7A,B).

### Proliferating cell nuclear antigen (PCNA) protein expression in PFC after transfection with Skp2 siRNA

**Figure 8:**
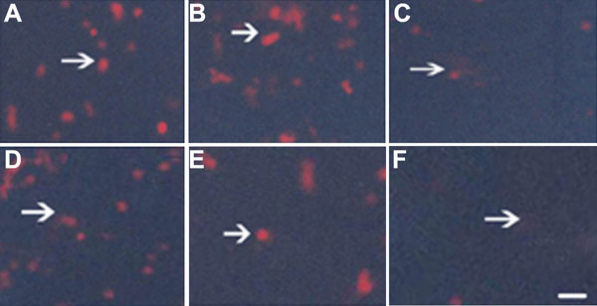
Down-regulation of PCNA protein by siRNA in PFC in vitro and in vivo. Immunofluorescence staining indicated the expression of PCNA decreased in PFC and NFC transfection with *Skp2* siRNA (**C**, **F**) when compared with hPFC and hNFC transfection with pSuppressor vehicle (**A**, **D**) or without transfection (**B**, **E**). The scale bar is equal to 40 μm.

**Figure 9:**
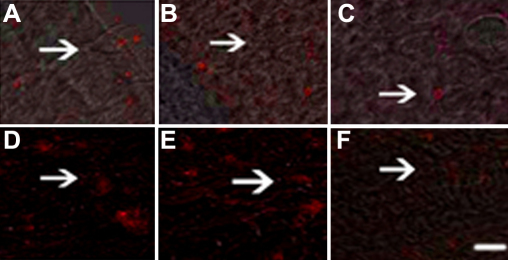
Down-regulation of PCNA protein by siRNA in PFC in vitro and in vivo. Immunofluorescence staining indicated the expression of PCNA decreased in PFC and NFC transfection with *Skp2* siRNA (**C**, **F**) when compared with hPFC and hNFC transfection with pSuppressor vehicle (**A**, **D**) or without transfection (**B**, **E**). The scale bar is equal to 40 μm.

PCNA is a marker that indicates the proliferation potential of cells. Immunofluorescence staining demonstrated expression of PCNA protein in the hPFC and hNFC transfected with pSuppressor vehicle (Figure 8A,D and Figure 9A,D) or without transfection (Figure 8B,E and Figure 9B,E) in vitro. Transfection with *Skp2* siRNA increased the expression of PCNA protein in cells of hPFC (Figure 8C,F and Figure 9C,F) in vitro.

### Histological observation for inhibition of PFC and NFC proliferation

**Figure 10:**
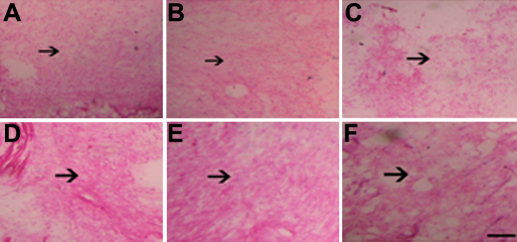
*Skp2* siRNA inhibited the proliferation of PFC and NFC in vivo. Hematoxylin and eosin staining was performed to examine the histological changes 14 days after transfection. Obvious PFC and NFC proliferation was detected in pSuppressor vehicle group or without transfection (**A**, **D**, **B**, **E**). There was little PFC and NFC proliferation in *Skp2* siRNA transfection group (**C**, **F**). Scale bar is equal to 20 μm.

High level of PFC and NFC proliferation can be detected in vehicle control (Figure 10A,D) and blank control (Figure 10B,E). However, PFC and NFC proliferation decreased in *Skp2* siRNA transfectant group (Figure 10C,F).

## Discussion

Pterygium is a condition characterized by epithelial overgrowth of the cornea, usually bilateral, and occupying a nasal interpalpebral location. It has a characteristic wing-shaped appearance. The pterygium epithelium that centripetally invades the cornea display squamous metaplasia and goblet cell hyperplasia. There is also an underlying breakdown of Bowman’s layer. A stromal overgrowth of fibroblasts and blood vessels is accompanied by an inflammatory cell infiltrate and an abnormal extracellular matrix accumulation composed of elastin and collagen [17-22].

Recently, we demonstrated expression of matrix metalloproteinases (MMPs) in resected pterygium specimens [23,24] and localized these enzymes at the advancing edges of the lesions [25]. In addition, data suggests that proinflammatory cytokines [26] can modify the expression of these extracellular matrix denaturing enzymes. These investigations imply that MMPs may play a significant role in tissue remodeling and invasion and in the dissolution of Bowman’s layer associated with pterygia.

Although MMPs may be important effective molecules in the pathogenesis of pterygia, the roles of cytokines and growth factors have yet to be established. Several studies have documented the expression of cytokines, such as tumor necrosis factor (TNF), basic fibroblast growth factor (bFGF), transforming growth factor (TGF), and platelet-derived growth factor (PDGF) in pterygia and cultured pterygium cells.

In addition, the localization of vascular endothelial growth factor (VEGF) in the pterygium epithelium and vascular endothelium and the presence of intraepithelial capillaries in pterygia suggest a role for angiogenic cytokines in this disease.

Cellular proliferation is regulated primarily by cell cycle regulation. Cell cycle progression is regulated by a combination of positive and negative regulators. Cyclin-dependent kinase (CDKs) inhibitors (CKI) negatively regulate progression of the cell cycle by inhibiting the activity of cyclin-CDK complexes. p27^kip1^, a member of the CKI family, plays a pivotal role in the control of cell proliferation [27-29]. The level of p27^kip1^ is high during the G_0_ phase but decreases rapidly upon reentry of the cells into the G_1_ phase. Rapid removal of p27^kip1^ at the G_0_/G_1_ transition is required for effective progression of the cell cycle to S phase [30-32]. According to our study, a low level of p27 ^kip1^ expression was correlated with high proliferative and migratory capacity, whereas nuclear accumulation of the Cyclin-dependent kinases inhibition (CKI) was associated with a quiesecnet and static phenotype. In previous studies, Yoshida et al. suggested that the disappearance of p27^kip1^ was correlated with cell proliferation in the corneal epithelium after injury [33].

The level of p27^kip1^ is regulated by Skp2. Skp2 is specifically required for p27^kip1^ ubiquitination and is a rate-limiting component of the machinery that degrades phosphorylated p27^kip1^. Skp2 is constitutively expressed in normal skin tissue and scar tissue. High expression of Skp2 and decreased expression of p27^kip1^ with fibroblast from pathological scar tissue [30]. There is negative correlation between expression of Skp2 and p27^kip1^ in fibroblasts from pathological scar tissue. However, there are no previous studies involving the role of Skp2 in hPFC proliferation after cataract surgery. After 48 h of transfection and 2 weeks of treatment with G418, transfection with *Skp2* siRNA decreased the expression of Skp2 protein in cells of hPFC by immunoflurescence staining and western blot. The above indicated that transfection with *Skp2* siRNA can inhibit expression of *Skp2* in hPFC in vitro and vivo. Then upregulation of p27^kip1^ of hPFC in vitro and in vivo was investigated by immunoflurescence staining and western blot. After transfection, we confirmed that the number of cells increased less in *Skp2* siRNA transfectant hPFC than vehicle control and blank control groups by MTT especially on 6th day. For BrdUrd incorporation assay, high level staining of BrdU can be detected in hLEC of vehicle control and blank control, but decreased in *Skp2* siRNA transfectant cells. Furthermore, for the first time, we showed that inhibition of *Skp2* expression by siRNA enhanced p27^kip1^ protein levels and prevented hPFC proliferation.

Approaches to inhibition the hPFC proliferation afer surgery by gene transfer have been reported by different research groups. Nelly et al. [34] showed that Trefoil Factor 1 can inhibit the proliferation of hPFC. Timothy et al. [20] confirmed that heparin-binding epidermal growth factor–like growth factor has the potential proliferative function for PFC. Juan et al. [35] showed that *P. peruviana* fruit juice can inhibition proliferation of PFC.

Finally, the number of cases included in this study is small and the result maybe has its limitation. In view of the growing interest in Skp2 and p27^kip1^ as a target for drug development in inhibition of hPFC proliferation after surgery, it is our hope that the data presented here will help to promote going efforts to decrease the recurrence of pterygium after surgery.
